# Advantage of Using of High-Sensitivity Troponin I Compared to Conventional Troponin I in Shortening Time to Rule out/in Acute Coronary Syndrome in Chest Pain Patients Presenting to the Emergency Department

**DOI:** 10.3390/medicina58101391

**Published:** 2022-10-04

**Authors:** Ziwei Lin, Patrizia Cardelli, Rossella Marino, Swee Han Lim, Salvatore Di Somma

**Affiliations:** 1 Emergency Medicine Department, National University Hospital, National University Health System, Singapore 119074, Singapore; 2 Department of Medical-Surgery Sciences and Translational Medicine, University Sapienza of Rome, 00189 Roma, Italy; 3 Department of Emergency Medicine, Singapore General Hospital, Singapore 169608, Singapore

**Keywords:** high-sensitivity troponin I, acute coronary syndrome, time to diagnosis, emergency department

## Abstract

*Background and Objectives*: We aimed to compare the time to diagnosis for acute coronary syndromes using high-sensitivity troponin I (hsTnI) and conventional troponin I (TnI) in patients presenting to the emergency department (ED) with chest pain. *Materials and Methods*: This was an observational prospective study involving patients presenting to the ED of Sant’Andrea Hospital University la Sapienza in Rome (Italy) with chest pain from January to December 2014. Serum troponin was drawn at presentation, and at 3, 6, 9, and/or 12 h if clinically indicated. Depending on date of recruitment, patients had either hsTnI (Abbott Laboratories) or TnI (Abbott Laboratories) performed. The primary endpoint was the time to diagnosis at index visit. *Results*: A total of 1059 patients were recruited, (673 [63.6%] male, median age 60 years [interquartile range 49–73 years]), out of whom 898 (84.8%) patients were evaluated with hsTnI and 161 (15.2%) with TnI. A total of 393 (37.1%) patients had the diagnosis of acute coronary syndrome in ED. The median time to diagnosis for those evaluated with TnI was 400 min, IQR 120–720 min, while the use of hsTnI led to a significantly shorter time to diagnosis (median 200 min, IQR 100–200 min, *p* < 0.001). *Conclusions*: This study confirms that in patients presenting to the emergency department with chest pain, the use of hsTnI is associated with a reduced time to ruling in/out ACS, and, consequently, hsTnI should be routinely used over TnI for more rapid identification of ACS with benefits for patients and related costs.

## 1. Introduction

Chest pain is a common complaint for patients presenting to the emergency department (ED). In the United States, more than 8 million patients presented to ED for chest pain in 2019 [1]. While chest pain accounts for approximately 10% of emergency department visits, only 10 to 20% of chest pain is diagnosed as cardiac chest pain [2], with a majority being attributed to a multitude of other diagnoses. Differentiating chest pain that originates from cardiac ischaemia, such as acute coronary syndrome (ACS), remains a diagnostic dilemma, as patients may present atypically. Conditions such as pulmonary embolism, aortic dissection, or pneumothorax may mimic ACS and present with symptoms such as chest pain and shortness of breath [3]. At time of presentation, approximately 80% of patients with chest pain do not have a clear diagnosis of ACS [4], with a further need to be monitored and worked up while in the emergency department or emergency observation units. While clinical evaluation of patients and repeated electrocardiograms (ECG) are essential, these alone are not enough to reliably rule out ACS, and have to be used in conjunction with other investigations [5].

The use of cardiac biomarkers, especially cardiac troponin, remains a cornerstone in the diagnosis of myocardial infarction [6]. The troponin complex consists of three subunits (troponin I, T, and C), and plays a vital role in regulating cardiac excitation and contraction [7]. In the setting of myocardial infarction, both troponin I and T are released from the damaged myocardium [8]. Conventional troponin assays are limited by their low sensitivities at the time of patient presentation, due to delay in the increase in circulating levels of troponin, requiring repeated testing 6–9 h after presentation [9], and are deemed as unlikely to be cost-effective [10].

With the introduction of high-sensitivity troponin, new protocols were generated to help in the risk stratification of patients presenting to the emergency department with chest pain or symptoms suggestive of cardiac ischaemia for myocardial infarction as quickly as within one to two hours [11,12]. This may ease the problem of overcrowding, and help with the appropriate allocation of limited ED resources. The current ESC guidelines suggest the possibility to reduce the time for ruling in/out ACS in ED using hsTnI [3]. Concurrently, the diagnosis of myocardial infarction may also be reached more quickly, leading to expedited treatment and disposition for patients with this condition. Hence, our study aims to compare the time to diagnosis between patients using high-sensitivity troponin I (hsTnI) and conventional troponin I (TnI) in differentiating between ACS and non-ACS chest pain in the ED.

## 2. Materials and Methods

This was an observational prospective study involving patients complaining of chest pain suggestive of ACS who presented to the ED of the study site, a 450-bed teaching hospital in Rome, Italy, from 1 January to 31 December 2014. Inclusion criteria were: patients aged between 18 to 90 years who presented with chest pain suggestive of ACS [13,14]. Patients with such symptoms were evaluated and serum troponin was drawn at presentation, with serial serum troponin performed at 3, 6, 9, and/or 12 h if clinically indicated. As during the study period, the institution transited from conventional troponin I (Abbott Laboratories, Chicago, IL, USA) to high-sensitivity troponin I (Abbott Laboratories, Chicago, IL, USA), patients either had high-sensitivity cardiac troponin I or conventional cardiac troponin I drawn, depending on when they were recruited.

The study was performed after approval from the Ethics Committee, according to the principles of the Helsinki Declaration. Informed consent was obtained from each patient. Data from patients were recorded and analysed using an identification code, allowing for anonymity. The following parameters were collected, including patient factors such as: age, gender, race, body mass index (BMI), past medical history, medications used, family history of cardiovascular disease, smoking history; factors regarding their presentation at ED, including: symptoms present, vital signs, ECG results, mode of transport to ED (via ambulance or autonomously), cardiovascular drugs administered while in ED, disposition (e.g., discharge, hospital admission to either general ward or high dependency or the intensive care unit), time to disposition, cost of visit per patient; as well as laboratory results such as serum troponin, glomerular filtration rate (GFR), glycaemia, and haemoglobin concentration.

Primary outcome was the time to diagnosis of ACS (comprising of STEMI, NSTEMI, and unstable angina) at index visit, as determined by the emergency physicians and confirmed by a cardiologist, as per the third universal definition of myocardial infarction.

### Statistical Analysis

Continuous data were reported as median, 25th, and 75th percentile, as well as means and standard deviation where appropriate. Categorical data were reported as frequency distributions. Continuous variables were analysed using Mann–Whitney U test and independent t-test as appropriate. Differences in categorical variables were analysed using χ^2^ test or Fisher’s exact test. Odds ratio (OR) and 95% confidence interval (95% CI) were reported. All tests were two-tailed, and statistical significance was set at 0.05. Statistical analyses were performed with SPSS V.20.0 for Windows statistical package (SPSS, Chicago, IL, USA).

## 3. Results

Our analysis includes a total of 1059 patients, out of whom 673 (63.6%) are male. The median age is 60 years, with an interquartile range (IQR) of 49 to 73 years. Out of our study population, 393 (37.1%) patients have ACS at index visit, among whom, 133 (12.6%) have the diagnosis of STEMI, 216 (20.4%) have NSTEMI, and 46 (4.3%) have unstable angina. A total of 664 (62.7%) patients have the final diagnosis of non-ACS chest pain.

Within our study population, 161 patients are evaluated using conventional troponin I (TnI) and 898 patients using high-sensitivity troponin I (hsTnI). For those with hsTnI, the median (IQR) for 0, 3, and 6 h hsTnI (pg/mL) are 6.00 (2.30–47.65), 5.30 (2.10–30.20), and 53.15 (2.68–707.58), respectively. For those with TnI performed, the median (IQR) for 0, 6, and 12 h TnI (ng/mL) are 0.010 (0.009–0.090), 0.010 (0.000–0.110), and 0.010 (0.000–0.030), respectively. The baseline characteristics of the hsTnI group and TnI group are similar, aside from ECG findings of sinus rhythm and T-wave inversions (Table 1, Table 2 and Table 3).

Among those patients evaluated using hsTnI, 331 (36.9%) have the primary outcome of ACS at index visit. Out of these patients, 110 (12.2%) have STEMI, 122 (13.6%) have NSTEMI, and 39 (4.3%) have unstable angina. For those who are evaluated using TnI, 64 (39.8%) have the primary diagnosis of ACS, out of whom 23 (14.3%) have STEMI, 34 (21.1%) have NSTEMI, and 7 (4.3%) have unstable angina. There is no significant difference in the incidence of ACS between the two groups (*p* = 0.485). 

The overall median time to diagnosis is 200 min (IQR 100 to 220 min) and the overall mean time to diagnosis is 245.2 min (SD 211.4 min). Comparing between patients evaluated with the use of hsTnI and TnI, the time to final diagnosis is significantly different (*p* < 0.001) with a median of 200 min (IQR 100–200 min) for patients where hsTnI is utilised versus a median of 400 min (IQR 120–720 min) for where TnI is used. The mean time to final diagnosis is also significantly shorter (*p* < 0.001) for the hsTnI group (mean 215 min, SD 175 min) as compared to the TnI group (mean 415 min, SD 298 min). 

For patients with the final diagnosis of non-ACS chest pain (*n* = 664, 62.7%), both the mean and median time to diagnosis is significantly shorter (*p* < 0.001) for those evaluated with hsTnI (mean 272.88 min, SD 183.16 min; median 200 min, IQR 200 to 240 min) as compared to TnI (mean 554.43 min, SD 242.13 min; median 720 min, IQR 380 to 720 min). 

For those with a diagnosis of NSTEMI, patients with hsTnI performed compared to TnI performed have a shorter mean time to final diagnosis (mean 146.57 min SD 74.79 min for hsTnI versus mean 258.82 min SD 219.09 min for TnI, *p* = 0.006). However, there is no significant difference for the median time to diagnosis between those evaluated with TnI and hsTnI for those with the final diagnosis of NSTEMI (median 150 min, IQR 80–200 min for hsTnI; median 135 min, IQR 62.5–360 min for TnI, *p* = 0.112)

**Figure 1:**
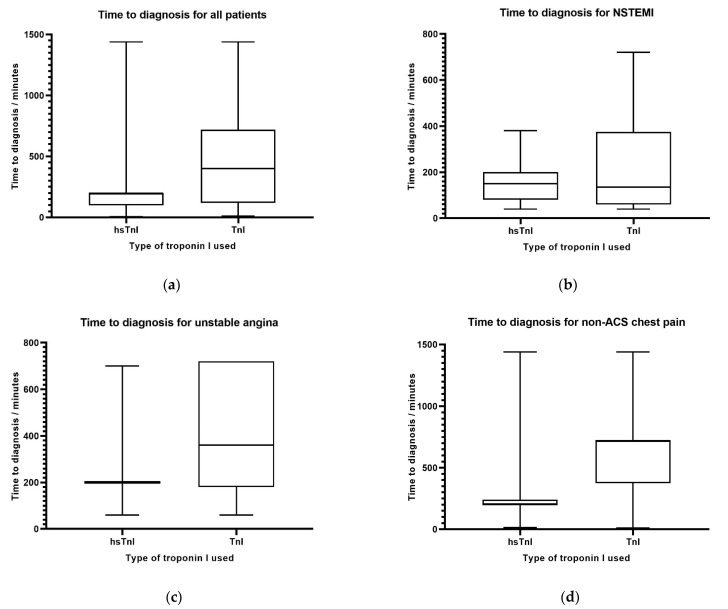
The difference in median time to diagnosis between hsTnI and cTnI for various final diagnoses: (**a**) time to diagnosis for all patients, (**b**) time to diagnosis for NSTEMI, (**c**) time to diagnosis for unstable angina, and (**d**) time to diagnosis for non-ACS chest pain.

For STEMI patients, the median time to diagnosis is similar (*p* = 0.460) between the hsTnI (median 15 min, IQR 10–20 min) and TnI groups (median 15 min, IQR 15–15 min). The mean time to diagnosis is also similar between the two groups (mean 25.75 min, SD 40.79 min for hsTnI; mean 71.48, SD 228.68 min for TnI, *p* = 0.349). There is also no significant difference for the time to diagnosis between those evaluated with TnI and hsTnI for those with the final diagnosis of unstable angina. The median time to diagnosis is 200 min (IQR 200–200 min) for those evaluated with hsTnI, and the median time to diagnosis is 360 min (IQR 187.5–550 min) for those evaluated with TnI, (*p* = 0.397). The mean time to diagnosis in the unstable angina group is 226.41 min (SD 137.93 min) for hsTnI and 373.57 min (SD 260.75 min) for TnI (*p* = 0.191). The findings for the median time to final diagnosis are summarised in Figure 1.

## 4. Discussion

In our study, we found that in patients who presented to the emergency department with symptoms suggestive of ACS, the time to diagnosis is significantly shorter when hsTnI is used as compared to TnI. This suggests that for patients with no ACS, the usage of hsTnI may allow us to identify those at low risk more effectively, and discharge them without the need for further investigations. For those presenting to the ED with chest pain, the median time to diagnosis is 200 min shorter for those evaluated with hsTnI as compared to those evaluated with TnI. These findings are in keeping with other studies that have illustrated that, when taken together with clinical context, high-sensitivity troponin may be able to rule out low-risk, non-cardiac chest pain more rapidly, allowing for earlier discharge from the emergency room for a select group of patients [3,12].

The median time to diagnosis of our group of undifferentiated chest pain patients is 200 min. This timing is similar to previous algorithms, which suggest 2 h as an appropriate time for assessment of patients with chest pain using high-sensitivity troponin. A 2 h algorithm by Reichlin et al. using high-sensitivity troponin T at baseline and 2 h was able to rule out myocardial infarction with a sensitivity of 96% and negative predictive value of 99.5%, as well as rule in myocardial infarction with a specificity of 99% and positive predictive value (PPV) of 85% [11]. A 1 h algorithm by Gimenez et al. utilizing high-sensitivity troponin I was able to rule out acute myocardial infarction with an NPV of 99.6% and rule in acute myocardial infarction with a PPV of 73.9% [15]. European Society of Cardiology (ESC) guidelines also recommend the use ‘rule-in’ and ‘rule-out’ algorithms at 1 h for high-sensitivity troponin (both T and I) in the diagnosis of NSTEMI in the emergency department [3]. The introduction of high-sensitivity troponin I assays are also shown to be associated with more rapid diagnosis and shorter median time spent in the emergency department [16]. In addition to reducing time to diagnosis, the usage of high-sensitivity troponin I over conventional troponin I is shown to achieve a mean reduction in length of stay of 6.2 h in patients presenting to the emergency department with undifferentiated chest pain [17].

For those with STEMI and unstable angina, although the mean time is shorter for those who had hsTnI performed, the difference is not significant when compared to those with TnI performed. This is unsurprising, as STEMI is an ECG diagnosis, and the diagnosis of unstable angina is made when there are symptoms suggestive of cardiac ischaemia in the absence of elevated troponin. While the usage of hsTnI may not significantly shorten the time to diagnosis for these patients, patients with these conditions remain a minority among those presenting to ED with chest pain (STEMI *n* = 133 (12.6%), unstable angina *n* = 46, (4.3%)).

In the United States of America, approximately 8,043,000 patients visited the emergency department for chest pain and related symptoms in 2019 [1]. A centre in the USA reported cost per patient bed-hour to be USD 58.20 in the emergency department [18]. With a mean reduction in time to diagnosis of 200.56 min for undifferentiated chest pain patients, this translates to a theoretical cost saving of USD 194.54 per patient and more than USD 1.5 billion annually. The usage of a 1 h algorithm with high-sensitivity cardiac troponin as compared to standard of care with conventional troponin shows a reduction in cost of up to 46%, with estimated total costs per patient of GBP 2480 for the 1 h algorithm compared to GBP 4561 for the standard of care group [19]. In Australia, the usage of high-sensitivity troponin I-supported algorithms increases diagnostic accuracy from 90.0% to 94.0%, with an average cost reduction per patient compared with standard care of AUD 490 [17].

In Sant’Andrea Hospital, within our study group, the median cost for each patient is shown to be significantly lower for those evaluated by hsTnI compared to TnI. For those with no ACS, the median cost for those evaluated by hsTnI is EUR 21,086 (IQR: EUR 20,031.70 to EUR 21,086) as compared to a median of EUR 75,909.60 (IQR: EUR 39,009.10 to EUR 75,909.60) for those evaluated with TnI. The trend is similar in those with NSTEMI (EUR 21,086 for hsTnI versus EUR 37,954.80 for TnI) and unstable angina (median: EUR 21,086 for hsTnI versus EUR 13,189.75 for TnI).

While the usage of high-sensitivity troponin may identify a group of patients with troponin elevation without myocardial infarction, elevated levels of high-sensitivity troponin among patients who present to ED with chest pain is associated with higher rates of mortality and MACE, regardless of index visit diagnosis [20,21]. In our study, we identified that patients in the hsTnI group actually have a higher attendance rate in the ED for chest pain within 1 year. We postulate that this may be as usage of hsTnI may reveal those with minute elevations in troponin that were previously not detectable, and would, hence, return to seek medical attention even without an initial diagnosis of ACS. High-sensitivity troponin should still be used over conventional troponin, as this allows identification of patients who are at high risk and should be followed up more closely, and may be considered of prognostic value for future events even in patients with detectable levels below the 99th percentile or with stable low-level elevations [22].

The use of high-sensitivity troponin is shown to be an effective and cost-effective means of ruling out AMI early [23]. The usage of high-sensitivity troponin may lead to more rapid diagnosis of patients with chest pain and earlier identification of those with acute myocardial infarction, and may lead to increased health cost savings [10]. While further studies are needed to come up with the best strategy and cut-offs for various high-sensitivity troponins, high sensitivity-troponin should be used in the emergency department for more expeditious diagnosis in the setting of chest pain.

### Limitations

This study is an observational study and, hence, prone to selection bias. It was also held in a single centre and, thus, results may not be generalizable to the international community. However, we tried to overcome this by having a large sample size (*N* = 1059). This study also does not look at the effect of usage of hsTnI over conventional TnI on long-term outcomes, such as future MACE or mortality (e.g., 30 days or 1 year).

## 5. Conclusions

The use of hsTnI is associated with a shorter time to final diagnosis among patients presenting to the emergency department with chest pain, and should be used over TnI. This allows for more rapid identification of those with ACS and better resource allocation in the emergency department.

## Figures and Tables

**Table 1:** Baseline characteristics of patients with hsTnI versus conventional TnI ^1^.

Variable	High-Sensitivity TnI (*n* = 898)	Conventional TnI (*n* = 161)
Gender		
Male	564 (62.8)	109 (67.7)
Female	334 (37.2)	52 (32.3)
Age	Median 60, IQR 50–73	Median 61, IQR 47–61
Race		
West European	839 (93.4)	149 (92.5)
East European	32 (3.6)	7 (4.3)
African	9 (1.0)	3 (1.9)
Hispanic	2 (0.2)	0 (0.0)
Asian	12 (1.3)	2 (1.2)
Past Medical History		
Family history of cardiovascular disease	166 (18.5)	26 (16.1)
Coronary artery disease	204 (22.7)	40 (24.8)
Ever-smoker	289 (32.2)	54 (33.5)
Hypercholesterolemia	331 (36.9)	53 (32.9)
Hypertension	520 (57.9)	82 (50.9)
Diabetes Mellitus	153 (17.0)	20 (12.4)
Final Diagnosis		
STEMI	110 (12.2)	23 (14.3)
NSTEMI	182 (20.3)	34 (21.1)
Unstable angina	39 (4.3)	7 (4.3)
Non-ACS chest pain	567 (63.1)	97 (60.2)

^1^ Data presented as *n* (%) unless otherwise specified.

**Table 2:** Laboratory results of patients with hsTnI versus conventional TnI ^1^.

Variable	High-Sensitivity TnI (*n* = 898)	Conventional TnI (*n* = 161)	*p*-Value
Creatinine (µmol/L)	0.93 (0.80–1.20)	0.90 (0.80–1.20)	0.728
Glomerular filtration rate ^2^	78.0 (60.0–94.0)	82.0 (63.0–97.5)	0.701
Random glucose (mg/dL)	107.0 (97.0–132.5)	104.0 (93.0–127.0)	0.072
Haemoglobin (g/L)	14.0 (13.0–15.0)	13.8 (12.5–15.0)	0.073
Creatine kinase (U/L)	84.0 (57.0–127.0)	86.5 (64.3–136.0)	0.599
Creatine kinase MB (U/L)	2.50 (1.40–5.55)	1.95 (1.37–7.25)	0.542
Myoglobin (ng/mL)	74.9 (51.0–325.8)	40.0 (33.1–41.3)	0.105
Uric acid (mg/dL)	5.5 (4.8–6.6)	4.6 (3.7–6.5)	0.227

^1^ Data are presented as median (IQR), ^2^ glomerular filtration rate as calculated by Modification of Diet in Renal Disease Study Equation (MDRD).

**Table 3:** Electrocardiogram (ECG) findings of study population ^1^.

Variable	High-Sensitivity TnI (*n* = 898)	Conventional TnI (*n* = 161)	*p*-Value
ST-elevation	104 (11.6%)	26 (16.1%)	0.104
ST-depression	72 (8.0%)	19 (11.8%)	0.115
New LBBB	11 (1.2%)	2 (1.2%)	1
T-wave inversions	73 (8.1%)	22 (13.7%)	0.024
Sinus rhythm	770 (85.7%)	122 (75.8%)	0.001
Atrial fibrillation	46 (5.1%)	5 (3.1%)	0.271
2nd/3rd degree AV block	2 (0.2%)	1 (0.6%)	0.391
Normal ECG	448 (49.9%)	67 (41.6%)	0.053
ECG equal to previous ECG	173 (19.3%)	30 (18.6%)	0.851

^1^ Data are presented as *n* (%).

## Data Availability

The data presented in this study are available on request from the corresponding author. The data are not publicly available due to patient confidentiality.
